# Taxonomy and Phylogeny of the Dileptid Ciliate Genus *Paradileptus* (Protista: Ciliophora), With a Brief Review and Redescriptions of Two Species Isolated From a Wetland in Northern China

**DOI:** 10.3389/fmicb.2021.709566

**Published:** 2021-09-21

**Authors:** Yong Chi, Zhe Wang, Borong Lu, Honggang Ma, Changjun Mu, Alan Warren, Yan Zhao

**Affiliations:** ^1^College of Life Sciences, Capital Normal University, Beijing, China; ^2^Institute of Evolution & Marine Biodiversity, and College of Fisheries, Ocean University of China, Qingdao, China; ^3^Weishan Special Aquaculture Base, Jining, China; ^4^Department of Life Sciences, Natural History Museum, London, United Kingdom

**Keywords:** morphology, *Paradileptus conicus*, *Paradileptus elephantinus*, Rhynchostomatia, ribosomal DNA

## Abstract

Members of the genus *Paradileptus* are apex predators in microbial food webs. They are often encountered in freshwater biotopes and have been used in research on water quality monitoring and ecology. Nevertheless, our understanding of the biodiversity of *Paradileptus*, especially its ecological and genetic diversities, is very poor which hinders our ability to understand the ecosystem services it provides. The present study gives a detailed account of two Chinese populations of *Paradileptus elephantinus* and *P. conicus* including their living morphology, infraciliature, and molecular phylogenies based on 18S, 5.8S, and ITS ribosomal DNA sequences. The phylogenetic relationships between these two species and other rhynchostomatians are investigated. We also explore the potential contribution of differentiation of the proboscis (e.g., extrusomes, dorsal brush, and differentiated kineties) to niche partitioning and speciation in *Paradileptus*. The global distribution of *Paradileptus* is summarized based on published data. Finally, a key to the identification of the valid species of *Paradileptus* is provided.

## Introduction

Ciliated protists (ciliates) are a diverse group of morphologically differentiated eukaryotic microorganisms that play critical roles in aquatic and terrestrial ecosystems by maintaining energy flow and nutrient cycles (Lynn, 2008; Song et al., 2009; Gao et al., 2016; Hu et al., 2019). Rhynchostomatians are a large group of raptorial ciliates with a conspicuous proboscis that bears well-developed extrusomes and a dorsal brush (Vd’ačný and Rajter, 2015; Vd’ačný et al., 2017). They occur in marine, limnetic, terrestrial, and anaerobic environments including both benthic and planktonic habitats (Lynn, 2008; Vd’ačný and Foissner, 2012). Nevertheless, our understanding of their biodiversity, especially their ecological and genetic diversities, is very poor which hinders our ability to understand the ecosystem services they provide.

According to the most recent classification of rhynchostomatians (Vd’ačný and Foissner, 2012), the subclass Rhynchostomatia Jankowski, 1980 contains three families and 12 genera. *Paradileptus* Wenrich, 1929 is a typically planktonic genus which is characterized by the obliquely truncated anterior end of the body with a broad peristomial field and a spiral proboscis that extends anteriorly (Wenrich, 1929; Foissner et al., 1999). Ten nominal species of *Paradileptus* have been reported but only four are valid, namely, *P. flagellatus* (Rousselet, 1890) Wenrich, 1929, *P. elephantinus* (Šveç, 1897) Kahl, 1931, *P. conicus* Wenrich, 1929, and *P. moniliger* (Ehrenberg, 1835) Vd’ačný & Foissner, 2012. Among these, only *P. conicus* has been studied using observations *in vivo*, protargol staining, and electron microscopy, and documented using photomicrographs (Foissner et al., 1995, 1999). However, there are still some characters that have not been investigated in detail, such as the shape and arrangement of extrusomes *in vivo*. Furthermore, the evolutionary relationships of *Paradileptus* remain unknown due to a lack of molecular data.

In the present study, we isolated two *Paradileptus* species (*P. elephantinus* and *P. conicus*) from freshwater habitats in Lake Weishan, northern China. The two species were investigated using observations *in vivo* and after protargol staining. Their molecular phylogenies inferred from 18S and ITS-5.8S rDNA sequences have been reconstructed. The global distribution pattern of *Paradileptus* is summarized based on previous and present studies. Finally, the classification of *Paradileptus* is updated and a key to the identification of the four valid species is supplied.

## Materials and Methods

### Sample Collection, Observation, and Identification

**FIGURE 1:**
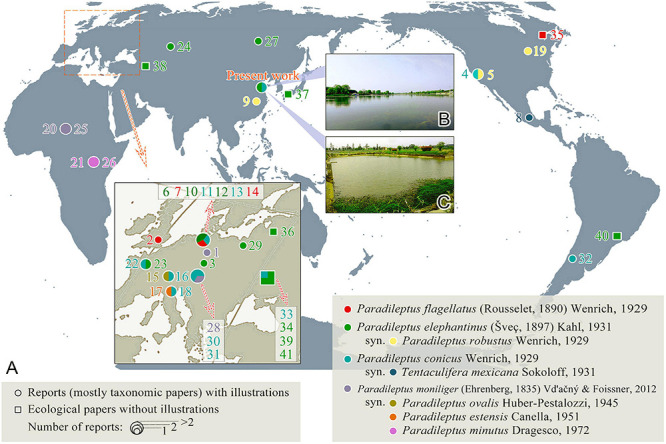
**(A)** The global geographic distribution of four valid *Paradileptus* species (see Table 1 for details). **(B)** The sampling location of *P. elephantinus* (Chinese population). **(C)** The sampling location of *P. conicus* (Chinese population).

*Paradileptus elephantinus* was collected from the Pontoon Dock of Lake Weishan Wetland Park (Figure 1B; N34°46′12″, E117°09′36″), Jining, China, on 24th April 2020. The physicochemical parameters of the sampling site were as follows: water temperature 17.8°C, atmospheric pressure 763.7 mm Hg, dissolved oxygen concentration 11.65 mg/L, salinity 0.63 ppt, and pH 9.39. *Paradileptus conicus* was isolated from an aquaculture pond of Weishan Special Aquaculture Base (Figure 1C; N34°46′18″, E117°09′54″), Jining, China, on 4th May 2020. The physicochemical parameters of the sampling site were as follows: water temperature 22.6°C, atmospheric pressure 754.8 mm Hg, dissolved oxygen concentration 7.11 mg/L, salinity 0.28 ppt, and pH 8.46. The two samples were collected from the water surface using a 20 μm mesh-sized plankton net and transferred into several Petri dishes for processing in the laboratory as soon as possible after collection (Bai et al., 2020).

Living cells were isolated with micropipettes and observed at 100–1000× magnifications using bright field and differential interference contrast microscopy (Olympus BX53) (Wu et al., 2020; Zhang et al., 2020). The infraciliature was revealed using the protargol staining method according to Wilbert (1975). Measurements and counts of stained specimens were conducted at magnifications of 100× and 1000×. Drawings of live cells were based on photomicrographs and free-hand sketches, while those of stained cells were accomplished with the help of a camera lucida at a magnification of 1000× (Lu et al., 2019). Terminology and systematics are mainly according to Vd’ačný et al. (2011a) and Vd’ačný and Foissner (2012).

### Geographical Distribution Analyses

The global distribution patterns of the *Paradileptus* species were mainly derived from previous reports that include morphological descriptions and recognizable illustrations. In addition, we selected several ecological reports in geographic regions not covered by morphological studies to show the range of *Paradileptus* distribution (Figure 1A and Table 1). In order to display the distribution of *Paradileptus* more accurately, we supply a list of the nominal species names as originally reported and the names by which they are now known based on the findings of the present study (Table 1). The literature used for this analysis was mainly derived from Vd’ačný and Foissner (2012).

**TABLE 1:** Global geographic distribution information of *Paradileptus* species.

**Number**	**Species name in original report**	**Current species name**	**Collection site**	**References**
1	*Amphileptus moniliger*	*P. moniliger*	Berlin, Germany	Ehrenberg, 1835
2	*Amphileptus flagellatus* Rousselet, 1890	*P. flagellatus*	London, United Kingdom	Rousselet, 1890
3	*Dileptus elephantinus* Šveç, 1897	*P. elephantinus*	Bohemia, Czechia	Šveç, 1897

4	*Paradileptus conicus* Wenrich, 1929	*P. conicus*	San Francisco, United States	Wenrich, 1929
5	*Paradileptus robustus* Wenrich, 1929	*P. elephantinus*		

6	*Paradileptus* (*Dileptus*) *elephantinus* (Šveç, 1897) Kahl, 1931	*P. elephantinus*	Germany	Kahl, 1931
7	*Paradileptus* (*Amphileptus*) *flagellatus* (Rousselet, 1890) Kahl, 1931	*P. flagellatus*		

8	*Tentaculifera mexicana* Sokoloff, 1931	*P. conicus*	Mexico City, Mexico	Sokoloff, 1931
9	*Paradileptus robustus* Wenrich, 1929	*P. elephantinus*	Nanjing, China	Wang and Nie, 1933

10	*Paradileptus elephantinus* Šveç, 1897	*P. elephantinus*	Hamburg, Germany	Kahl, 1935
11	*Paradileptus conicus* Wenrich, 1929	*P. conicus*		

12	*Paradileptus elephantinus* Šveç	*P. elephantinus*	Germany	Kahl, 1943
13	*Paradileptus conicus* Wenrich	*P. conicus*		
14	*Paradileptus flagellatus* Rousselet	*P. flagellatus*		

15	*Paradileptus ovalis* Huber-Pestalozzi, 1945	*P. moniliger*	Lake Zurich, Switzerland	Huber-Pestalozzi, 1945
16	*Paradileptus conicus*	*P. conicus*		

17	*Paradileptus estensis* Canella, 1951	*P. moniliger*	Ferrara, Italy	Canella, 1951
18	*Paradileptus conicus* Wenrich	*P. conicus*		

19	*Paradileptus robustus* Wenrich	*P. elephantinus*	Michigan, United States	Lundin and West, 1963
20	*Paradileptus elephantinus* Šveç	*P. moniliger*	Chari River, Chad	Dragesco, 1972a
21	*Paradileptus minutus* Dragesco, 1972	*P. moniliger*	Kasinga Channel, Uganda	Dragesco, 1972b

22	*Paradileptus conicus* Wenrich, 1929	*P. conicus*	France	Fryd-Versavel et al., 1975
23	*Paradileptus elephantinus* Šveç, 1897	*P. elephantinus*		

24	*Paradileptus elephantinus* Šveç, 1897	*P. elephantinus*	Volga River, Russia	Mamaeva, 1979

25	*Paradileptus elephantinus* Šveç	*P. moniliger*	Chari River, Chad	Dragesco and Dragesco-Kernéis, 1986
26	*Paradileptus minutus* Dragesco, 1972	*P. moniliger*	Kasinga Channel, Uganda	

27	*Paradileptus elephantinus* Šveç, 1897	*P. elephantinus*	Baikal Lake area, Russia	Lokot’, 1987
28	*Paradileptus elephantinus* Šveç, 1897	*P. moniliger*	Styria, Austria	Krainer, 1988
29	*Paradileptus elephantinus* Šveç, 1897	*P. elephantinus*	Wigry National Park, Poland	Czapik and Fyda, 1995
30	*Paradileptus elephantinus* (Šveç, 1897) Kahl, 1931	*P. conicus*	Salzburg, Austria	Foissner et al., 1995
31	*Paradileptus elephantinus* (Šveç, 1897) Kahl, 1931	*P. conicus*	Salzburg, Austria	Foissner et al., 1999
32	*Paradileptus elephantinus* (Šveç)	*P. conicus*	Patagonia, Argentina	Modenutti and Pérez, 2001

33	*Paradileptus conicus* Wenrich	P. conicus*	Ukraine	Kravchenko, 1969
34	*Paradileptus elephantinus*	P. elephantinus*		

35	*Paradileptus flagellatus* (Rousselet)	P. flagellatus*	Quebec, Canada	Puytorac et al., 1972
36	*Paradileptus elephantinus* (Šveç)	P. elephantinus*	Latvia	Liepa, 1983
37	*Paradileptus elephantinus*	P. elephantinus*	Hiroshima and Higashi-Hiroshima, Japan	Matsuoka et al., 1983
38	*Paradileptus elephantinus* Šveç	P. elephantinus*	Azerbaijan	Alekperov, 1984
39	*Paradileptus elephantinus* (Šveç)	P. elephantinus*	Ukraine	Oleksiv, 1985
40	*Paradileptus elephantinus*	P. elephantinus*	Sao Paulo, Brazil	Barbieri and Godinho-Orlandi, 1989
41	*Paradileptus elephantinus* Šveç	P. elephantinus*	Ukraine	Nebrat, 1992

*Since there is no illustration, the name in the original report is used.

### DNA Extraction, Polymerase Chain Reaction (PCR) Amplification, and Sequencing

For each species, a single cell was isolated from the original sample and washed five times with 0.22 μm filtered *in situ* water to remove potential contaminants. Genomic DNA was extracted from the cleaned cells using the DNeasy Blood and Tissue Kit (QIAGEN, Hilden, Germany) following the manufacturer’s instructions but modified by using 1/4 of the suggested volume for each solution. Q5^®^ Hot Start High-Fidelity 2× Master Mix DNA polymerase (New England BioLabs) was used to amplify the 18S and ITS-5.8S rDNA using universal eukaryotic primers 82F (5′-GAAACTGCGAATGGCTC-3′) and ITS-R (5′-TACTGATATGCTTAAGTTCAGCGG-3′) (Sogin, 1989; Chi et al., 2021). PCR amplifications were performed according to the following procedure: initial denaturation at 98°C for 30 s, followed by 18 cycles of amplification (98°C, 10 s; 69–51°C touchdown, 30 s; 72°C, 1 min), and another 18 cycles (98°C, 10 s; 51°C, 30 s; 72°C, 1 min), with a final extension of 72°C for 5 min (Lian et al., 2020). PCR products were sequenced bidirectionally in Tsingke Biological Technology Company (Qingdao, China) and assembled by SeqMan (DNAStar).

### Phylogenetic Analyses

All available 18S, 5.8S, and ITS rDNA sequences of free-living litostomateans from known morphospecies were downloaded from the GenBank database and were compiled into four datasets each of which was used for separate phylogenetic analyses. The first dataset included 83 18S rDNA sequences of *P. elephantinus* and *P. conicus*, their related rhynchostomatians, other litostomateans, and armophoreans (outgroup taxa) and was used to construct the 18S tree of the Litostomatea. The second dataset contained 40 18S rDNA sequences of rhynchostomatians and spathidiids and was used to generate the phylogenetic tree focusing on the subclass Rhynchostomatia. The third dataset comprising 26 5.8S and ITS rDNA sequences of the two *Paradileptus* species, all available rhynchostomatians, and five spathidiids (outgroup taxa) was used to construct the ITS-5.8S tree focusing on the subclass Rhynchostomatia. The 18S, 5.8S, and ITS rDNA sequences of the rhynchostomatians and spathidiids were concatenated by SeaView v4 (Gouy et al., 2009) to form the fourth dataset that was used to generate the concatenated tree. See Supplementary Table 1 for sequence sources of these datasets.

Initial alignments were performed using the MAFFT algorithm with default parameters on the web server GUIDANCE2^1^ (Zhang et al., 2019). Columns with scores less than 95% and residues with scores less than 90% were filtered, thus rendering the alignment suitably refined for the phylogenetic analysis. Maximum likelihood (ML) analysis was performed with RAxML-HPC2 on XSEDE v.8.2.12 (Stamatakis, 2014) on the CIPRES Science Gateway (Miller et al., 2010). Bayesian inference (BI) analysis was performed with MrBayes on XSEDE v.3.2.7a (Ronquist et al., 2012; Wang et al., 2019) on the CIPRES Science Gateway using the GTR + I + G evolutionary model as the best-fit model selected by MrModeltest v.2.2 (Nylander, 2004) according to the Akaike Information Criterion (AIC). Markov chain Monte Carlo (MCMC) simulations were then run with two sets of four chains using the default settings for 10,000,000 generations, with a sample frequency of 100 generations. The first 10% of trees were discarded as burn-in. All remaining trees were used to calculate the posterior probabilities. TreeView v.1.6.6 (Page, 1996) and MEGA v.5.2 (Tamura et al., 2011) were used to visualize tree topologies. BioEdit (Hall, 1999) was used to analyze the genetic difference between the two *Paradileptus* sequences.

## Results

Class Litostomatea Small & Lynn, 1981
Subclass Rhynchostomatia Jankowski, 1980
Order Dileptida Jankowski, 1978
Family Dileptidae Jankowski, 1980
Genus *Paradileptus* Wenrich, 1929

Wenrich (1929) established the genus *Paradileptus* with descriptions of two new species (*P. conicus* and *P. robustus*) and transferred *Amphileptus flagellatus* Rousselet, 1890 into it as the type species. However, *Amphileptus moniliger* Ehrenberg, 1835 was the first reported species, although it was only recently transferred into *Paradileptus* (Vd’ačný and Foissner, 2012). To date, there have been more than 30 taxonomic reports on *Paradileptus* with 10 nominal species established, namely, *P. flagellatus* (Rousselet, 1890) Wenrich, 1929, *P. elephantinus* (Šveç, 1897) Kahl, 1931, *P. robustus* Wenrich, 1929, *P. conicus* Wenrich, 1929, *P. ovalis* Huber-Pestalozzi, 1945, *P. estensis* Canella, 1951, *P. minutus* Dragesco, 1972, *P. moniliger* (Ehrenberg, 1835) Vd’ačný & Foissner, 2012, *P. caducus* Kahl, 1935, and *P. canellai* Dragesco, 1966. According to Vd’ačný and Foissner (2012), only one species is valid, i.e., *P. elephantinus*, whereas the findings of the present study support the validity of two species, i.e., *P. elephantinus* and *P. conicus*. Furthermore, our analysis of the literature suggests that *P. flagellatus* and *P. moniliger* are also probably valid. Consequently, we recognize four species, i.e., *P. flagellatus*, *P. elephantinus*, *P. conicus*, and *P. moniliger*. Traditionally, the establishment of species in this genus is based on living characters, the infraciliature having been rarely reported (Fryd-Versavel et al., 1975; Foissner et al., 1999). These species therefore need to be redefined based on a combination of *in vivo* morphological and infraciliature data. Furthermore, evolutionary relationships within *Paradileptus* remain unresolved due to the lack of molecular information.

### Improved Diagnosis of the Genus *Paradileptus*

Flexible but non-contractile planktonic dileptids; body trunk usually ovoidal or conical; oral field broad, dish-like, and prolonged anteriorly into a spiral proboscis; contractile vacuoles small and numerous, distributed throughout body; two types of extrusomes attached to proboscis oral bulge; somatic kineties difficult to recognize, somatic kinetosomes of body trunk loosely arranged; dorsal brush diffuse and staggered; right side of circumoral kinety accompanied by a perioral kinety, left side by numerous oblique preoral kineties; freshwater habitat.

### Type Species

*Amphileptus flagellatus* Rousselet, 1890.

### Species Distributions

*Paradileptus* is seemingly cosmopolitan having been recorded from 21 countries representing five continents (Africa, Asia, Europe, North America, and South America). It has been reported most frequently in Europe but has not been found in Antarctica or Oceania. It mainly occurs in freshwater habitats such as lakes, reservoirs, ponds, and rivers.

The abundances of the two species reported here differed significantly, i.e., there were about 20 cells of *Paradileptus conicus* per 10 ml but only about two cells of *P. elephantinus* per 10 ml. Nevertheless, both species show a wide distribution and have been reported from four continents (Asia, Europe, North America, and South America). *Paradileptus moniliger*, the earliest reported species in the genus, has only been recorded in Africa and Europe, and *P. flagellatus* only in Europe and North America (Figure 1A and Table 1).

### *Paradileptus elephantinus* (Šveç, 1897) Kahl, 1931

#### Synonyms

This list is adapted from that originally compiled by Vd’ačný and Foissner (2012).

1897 *Dileptus elephantinus* n. sp.–Šveç, Bulletin International de I’Academie des Sciènces 4: 13, 14 [Figures 13, 14] (original description).
1929 *Paradileptus robustus* n. sp.–Wenrich, Transactions of the American Microscopical Society 48: 357–359 [Figure 5] (detailed description based on living cells, synonymy proposed by Kahl, 1931).
1931 *Paradileptus* (*Dileptus*) *elephantinus* (Šveç, 1897)–Kahl, Die Tierwelt Deutschlands 21: 210 [Figure 24 on page 206] (short revision with author combination).
1933 *Paradileptus robustus* Wenrich, 1929–Wang and Nie, Contributions from the Biological Laboratory of the Science Society of China 10: 31–33 [Figure 27] (redescription of morphology based on living cells).
1935 *Paradileptus elephantinus* Šveç, 1897–Kahl, Die Tierwelt Deutschlands 30: 823 [Figures 18, 19 on page 808] (brief review and description).
1943 *Paradileptus elephantinus* Šveç–Kahl, Infusorien: 32 [Figure 3 on page 30] (short review).
1963 *Paradileptus elephantinus*–Lundin and West, Northern Michigan College Press: 1–175 (a Michigan population with illustration).
1975 *Paradileptus elephantinus* Šveç, 1897–Fryd-Versavel et al., Protistologica 11: 520–521 [Figures 18A,B] (morphological redescription, including infraciliature information).
1979 *Paradileptus elephantinus* Šveç, 1897–Mamaeva, Nauka: 31 (brief review and ecology, with illustration).
1987 *Paradileptus elephantinus* Šveç, 1897–Lokot’, Nauka: 35 (ecological report with illustration).
1995 *Paradileptus elephantinus* Šveç, 1897–Czapik and Fyda, Przegląd Zoologiczny 39: 65–72 (ecological report with illustration).

*Paradileptus elephantinus* was originally reported by Šveç (1897) under the name *Dileptus elephantinus*. Subsequently, this organism was reported numerous times, especially in ecological works but, with the exception of the study by Fryd-Versavel et al. (1975), details of its living characters and infraciliature were not provided. Based on both previous and present studies, an improved diagnosis is supplied.

#### Improved Diagnosis

Body about 180–600 × 100–350 μm *in vivo*; trunk oval in outline, with anterior spiral proboscis; macronucleus moniliform, micronuclei closely associated with macronuclear nodules; numerous contractile vacuoles distributed throughout body; two size-types of rod-shaped extrusomes attached to proboscis oral bulge; cortical granules colorless, oblong and densely scattered throughout cortex; dorsal brush diffuse and staggered; about 83–112 preoral kineties; freshwater habitat.

#### Voucher Slides

Three voucher slides with protargol-stained specimens are deposited in the Laboratory of Protozoology, Ocean University of China (OUC) with registration numbers: CY2020042401-01, 02, 03.

#### Morphological Description of Chinese Population

**FIGURE 2:**
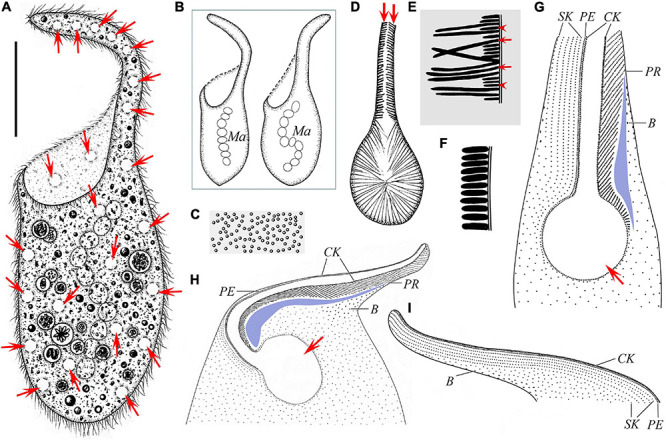
Schematic drawings of *Paradileptus elephantinus* from life **(A–F)** and after protargol staining **(G–I)**. **(A)** Ventral view of a typical individual; arrows mark the contractile vacuoles. **(B)** Two individuals to show the different body shapes and distribution of macronuclear nodules. **(C)** Cortical granules underneath the pellicle. **(D)** Detail of oral apparatus, showing the oral opening, the basket supported by fibers, and the rod-shaped type II extrusomes (arrows) attached to the proboscis oral bulge. **(E)** Schematic drawing of a tangential optical section of the proboscis; arrows mark the elongated rod-shaped type I extrusomes; arrowheads indicate the short rod-shaped type II extrusomes. **(F)** Schematic drawing of a cross-section of the cortex showing the densely arranged oblong cortical granules. **(G)** Detail of oral region showing the circular oral opening (arrow), glabrous area (blue block), recognizable somatic kineties, perioral kinety, circumoral kinety, dorsal brush, and obliquely oriented preoral kineties. **(H,I)** Ciliary pattern in ventral view **(H)** and dorsal view **(I)** of anterior body portion of the same individual; arrow in panel **(H)** marks the oral opening. B, dorsal brush; CK, circumoral kinety; Ma, macronucleus; PE, perioral kinety; PR, preoral kineties; SK, somatic kineties. Scale bar = 70 μm **(A)**.

**FIGURE 3:**
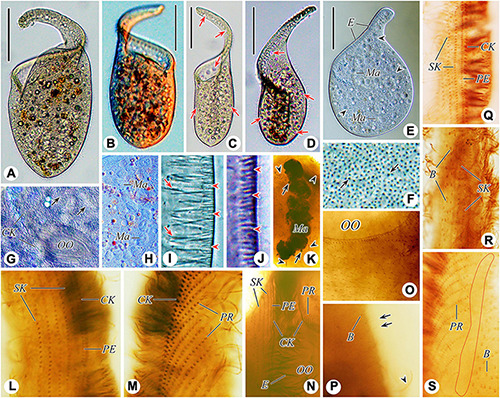
Photomicrographs of *Paradileptus elephantinus* from life **(A–J)** and after protargol staining **(K–S)**. **(A–D)** Various individuals to show different body shapes; arrows indicate the contractile vacuoles. **(E)** To show the rod-shaped extrusomes regularly arranged in the proboscis and developing extrusomes scattered in the cytoplasm (arrowheads). **(F)** Cortical granules (arrows) underneath the cortex. **(G)** Detail of oral area showing the densely arranged cortical granules (arrows). **(H)** Detail of the moniliform macronucleus. **(I)** Tangential optical section of proboscis to show the elongated rod-shaped type I extrusomes (arrows) and short rod-shaped type II extrusomes (arrowheads). **(J)** Tangential optical section of cortex to show the oblong cortical granules (arrowheads). **(K)** Details of the macronucleus, micronuclei (arrows), and developing extrusomes (arrowheads). **(L,M)** Right **(L)** and left **(M)** views of the anterior regions of the proboscis of the same individual showing the perioral kinety, circumoral kinety, and recognizable somatic kineties. **(N)** Detail of proximal portion of oral apparatus. **(O)** Showing the loosely arranged somatic kinetosomes. **(P)** Left view of posterior portion of proboscis; arrows indicate the monokinetidal tails of dorsal brush with bristles; arrowhead marks a somatic cilium. **(Q,R)** Right view of the anterior **(Q)** and middle **(R)** regions of the proboscis. **(S)** Detail of the posterior portion of the proboscis; red circle indicates the glabrous area. B, dorsal brush; CK, circumoral kinety; E, extrusomes; Ma, macronucleus; OO, oral opening; PE, perioral kinety; PR, preoral kineties; SK, somatic kineties. Scale bars = 90 μm **(A,B,D,E)**, 130 μm **(C)**.

Body about 265–420 × 85–200 μm *in vivo* when swimming spirally (natural form), about 315 × 120 μm on average, length to width ratio about 2.1–3.6:1; 320–534 × 107–220 μm in protargol-stained specimens; flexible and non-contractile, trunk usually oval in outline, obliquely truncated anteriorly with a disk-like oral field and a prolonged helical proboscis, rounded or slightly pointed posteriorly; proboscis conspicuous and highly variable in length, easily damaged due to its fragility (Figures 2A,B, 3A–D, and Table 2). Macronucleus moniliform with 8–14 nodules, about 11 nodules on average, each nodule about 23 × 18 μm in size, located in trunk; micronuclei not detected *in vivo* and inconspicuous in protargol-stained specimens, ovoidal or globular (about 3.5 μm in diameter, *n* = 8), closely associated with macronuclear nodules (Figures 2A,B, 3E,H,K). Contractile vacuoles small and numerous, about 6.6–10.0 μm in diameter, distributed throughout proboscis and trunk (Figures 2A, 3C,D). Two types of rod-shaped extrusomes regularly distributed in proboscis oral bulge: type I, 11.6–13.4 μm long *in vivo*, on average about 12.3 μm; type II, 3.1–3.7 μm long *in vivo*, on average about 3.4 μm; developing argentophilic extrusomes scattered in cytoplasm, 9.2–12.6 μm long *in vivo*, on average about 10.5 μm (Figures 2D,E, 3E,I,K,N). Pellicle flexible and thin with numerous oblong, colorless cortical granules, about 2.6 × 0.7 μm in size, scattered throughout cortex and thus not forming oblique rows, as usual of other dileptids (Figures 2C,F, 3F,G,J). Cytoplasm brownish at low magnifications, with numerous cytoplasmic granules and food vacuoles, without symbiotic green algae (Figures 2A, 3A–E). Locomotion by swimming while rotating about main body axis.

**TABLE 2:** Morphometric data of Chinese populations of *Paradileptus elephantinus* (upper line) and *P. conicus* (lower line).

**Character**	**Min**	**Max**	**Mean**	**M**	**SD**	**CV**	**N**
Body, length* (μm)	265	420	317.0	305	53.9	17.0	5
	165	230	192.9	185	21.5	11.2	7
Body, width* (μm)	85	200	122.0	100	41.1	33.7	5
	65	100	78.6	75	11.9	15.1	7
Oral apparatus, length* (μm)	130	210	164.0	165	26.3	16.1	5
	70	125	98.6	105	16.8	17.1	7
Body, length** (μm)	320	534	426.3	431	62.0	14.5	19
	118	273	207.3	202	39.1	18.8	21
Body, width** (μm)	107	220	162.8	172	35.2	21.6	19
	45	116	73.5	71	20.2	27.4	21
Oral opening, length** (μm)	49	87	64.4	59	14.1	21.9	7
	20	29	23.7	24	2.9	12.2	11
Oral opening, width** (μm)	38	71	48.6	44	10.8	22.3	7
	12	23	17.5	16	3.7	21.2	11
Preoral kineties, number	83	112	95.3	96	9.7	10.1	7
	60	85	66.7	65	6.8	10.2	11
Ma nodules, number	8	14	10.7	11	2.0	18.8	9
	6	17	10.2	10	2.4	23.1	21
Ma nodule, length** (μm)	16	30	22.9	22	4.6	20.1	9
	11	25	16.7	16	3.6	21.6	21
Ma nodule, width** (μm)	13	26	18.2	19	4.1	22.5	9
	7	14	10.7	10	2.0	19.0	21
Oral fibers, length*** (μm)	9.7	15.0	12.3	12.2	1.4	11.2	19
	7.4	13.6	10.2	9.8	1.8	17.5	19
Type I extrusomes in proboscis oral bulge, length*** (μm)	11.6	13.4	12.3	12.3	0.5	4.4	8
	4.0	5.7	5.0	5.0	0.5	9.0	19
Type II extrusomes in proboscis oral bulge, length*** (μm)	3.1	3.7	3.4	3.4	0.2	5.2	13
	1.7	2.3	2.0	2.0	0.2	8.8	9
Developing extrusomes in cytoplasm, length*** (μm)	9.2	12.6	10.5	10.4	0.9	8.4	11
	3.2	4.4	3.7	3.6	0.4	9.6	9
Cortical granules, length*** (μm)	2.2	2.9	2.6	2.7	0.2	8.7	9
	1.3	1.7	1.5	1.5	0.1	6.4	13
Cortical granules, width*** (μm)	0.5	0.9	0.7	0.7	0.1	19.7	12
	0.6	0.8	0.7	0.7	0.1	8.0	11
Contractile vacuoles, diameter*** (μm)	6.6	10.0	8.3	8.1	1.1	12.9	19
	6.9	9.5	8.4	8.8	0.7	8.8	19
Bristles, length** (μm)	3.0	4.0	3.3	3.2	0.2	7.3	15
	1.9	3.4	2.5	2.4	0.5	20.2	12

*CV, coefficient of variation in %; M, Median; Ma, macronucleus; Max, maximum; Mean, arithmetic mean; Min, minimum; N, number of specimens investigated; SD, standard deviation.*

*Data based on living cells while swimming.

**Data based on protargol-stained specimens.

***Data based on squashed living cells. Ma nodules for measurement were selected randomly in each individual.

Somatic cilia 10–14 μm long *in vivo* and widely spaced. Somatic kineties difficult to recognize due to somatic kinetosomes (monokinetids) loosely arranged in body trunk; only about 4–7 recognizable somatic kineties on right side of perioral kinety, starting at anterior of proboscis, extending posteriad parallel to perioral kinety and terminating at trunk; somatic kinetosomes progressively loosely arranged from anterior to posterior, becoming unrecognizable near oral opening (Figures 2G–I, 3L,N,O,Q). Circumoral kinety with closely spaced kinetosomes, distributed along contour of oral bulge in a basically U-shaped pattern, composed of dikinetids in proboscis and monokinetids around oral opening (Figures 2G–I, 3L–N,Q). Perioral kinety (first kinety on right side of circumoral kinety) with closely spaced monokinetids, commencing at anterior end of proboscis and extending to proximal part of oral opening; perioral kinety closer to circumoral kinety than to first right somatic kinety (Figures 2G–I, 3L,N,Q). Eighty-three to 112 oblique preoral kineties on left side of circumoral kinety, progressively shortened from middle of proboscis to both ends, that is, middle kineties composed of about 30–35 narrowly spaced monokinetids, fewer kinetosomes in each successive kinety (Figures 2G,H, 3M,N). Kinetosomes of dorsal brush diffuse and scattered throughout proboscis (Figures 2G–I, 3P,R,S). Dorsal brush also containing difficult-to-recognize monokinetidal tails with bristles about 3.0–4.0 μm long and extending to base of proboscis (Figure 3P). Type of dorsal brush bristles not discernable in protargol-stained specimens. Glabrous area on left of preoral kineties, extending to proximal part of oral opening (Figures 2G,H, 3S).

Oral apparatus large, consisting of a helical proboscis and a dish-like field at anterior end of trunk; oral region occupies about 45–55% of body length, distance from anterior end of proboscis to oral opening about 130–210 μm (Figures 2A,B, 3A–D). Oral opening elliptical to circular, about 49–87 × 38–71 μm in protargol-stained specimens, located laterally and inverted; oral region surrounded by circumoral kinety; perioral kinety on right side of circumoral kinety, preoral kineties on left side (Figures 2D,G,H, 3G). Pharyngeal basket conspicuous, composed of numerous fibers, about 9.7–15.0 μm long *in vivo* (Figures 2D, 3G).

### *Paradileptus conicus* Wenrich, 1929

#### Synonyms

This list is adapted from that originally compiled by Vd’ačný and Foissner (2012).

**FIGURE 4:**
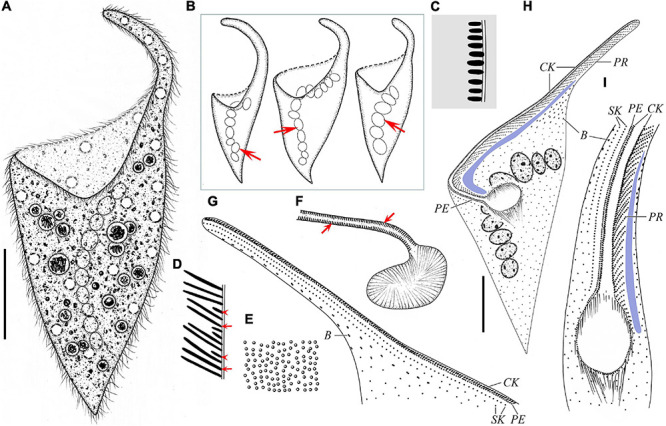
Schematic drawings of *Paradileptus conicus* from life **(A–F)** and after protargol staining **(G–I)**. **(A)** Ventral view of a typical individual. **(B)** Three individuals to show different body shapes and the moniliform macronucleus (arrows). **(C)** Schematic drawing of a cross-section of the cortex showing the oblong cortical granules. **(D)** Schematic drawing of a tangential optical section of the proboscis; arrows mark the rod-shaped type I extrusomes; arrowheads indicate the oblong type II extrusomes. **(E)** Cortical granules underneath the pellicle. **(F)** Detail of oral apparatus, showing the oral opening, the basket supported by fibers, and the oblong type II extrusomes (arrows) attached to the proboscis oral bulge. **(G,H)** Ventral **(H)** and dorsal **(G)** views of the same individual showing the ciliary pattern. **(I)** Oral region showing the oral opening, glabrous area (blue block), recognizable somatic kineties, perioral kinety, circumoral kinety, dorsal brush, and obliquely oriented preoral kineties. B, dorsal brush; CK, circumoral kinety; PE, perioral kinety; PR, preoral kineties; SK, somatic kineties. Scale bars = 40 μm **(A,H)**.

**FIGURE 5:**
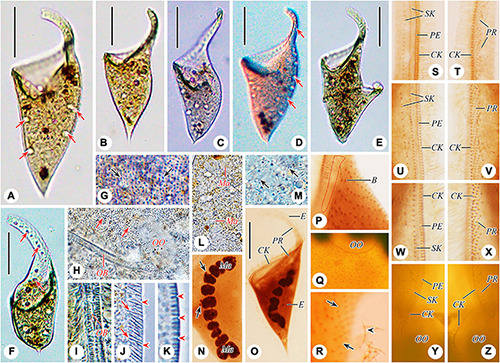
Photomicrographs of *Paradileptus conicus* from life **(A–M)** and after protargol staining **(N–Z)**. **(A–D)** Various individuals to show different body shapes, arrows indicate the contractile vacuoles. **(E)** Cell undergoing binary fission. **(F)** A squashed cell to show the numerous contractile vacuoles (arrows mark three of these). **(G)** Cortical granules (arrows) underneath the pellicle. **(H)** Detail of oral region showing the densely arranged cortical granules (arrows). **(I)** Detail of the proboscis oral bulge. **(J)** Tangential optical section of the proboscis to show the rod-shaped type I extrusomes (arrows) and oblong type II extrusomes (arrowheads). **(K)** Tangential optical section of the cortex to show the oblong cortical granules (arrowheads). **(L,N)** Detail of the macronucleus and micronuclei (arrows). **(M)** Developing extrusomes scattered in the cytoplasm (arrows). **(O)** Dorsal view showing the circumoral kinety, developing extrusomes and preoral kineties. **(P,R)** Detail of the posterior portion of the proboscis; red circle indicates the glabrous area; arrows indicate the monokinetidal tails of dorsal brush with bristles; arrowhead marks a somatic cilium. **(Q)** Showing the loosely arranged somatic kinetosomes. **(S–Z)** Right **(S,U,W,Y)** and left **(T,V,X,Z)** views of the anterior (**S,T** from the same cell), middle (**U,V** from the same cell), and posterior (**W,X** from the same cell) regions of the proboscis, and proximal portion of oral apparatus (**Y,Z** from the same cell). B, dorsal brush; CK, circumoral kinety; E, extrusomes; Ma, macronucleus; OB, oral bulge; OO, oral opening; PE, perioral kinety; PR, preoral kineties; SK, somatic kineties. Scale bars = 60 μm **(A,C–E)**, 50 μm **(B,F,O)**.

**FIGURE 6:**
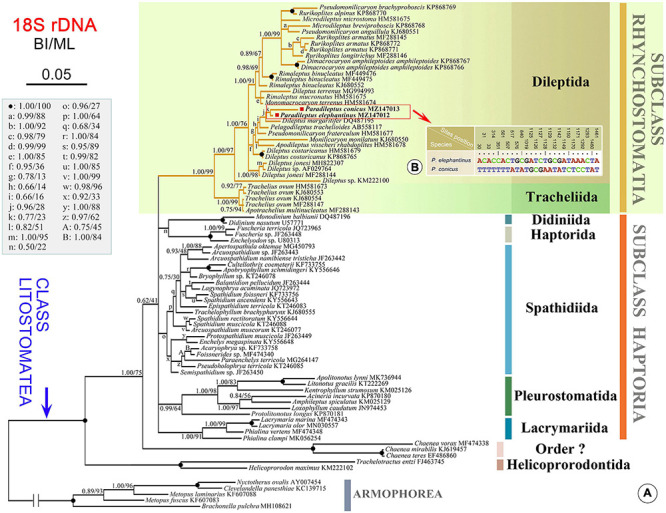
**(A)** Bayesian inference (BI) tree inferred from 18S rDNA sequences (78 litostomatean and five armophorean taxa). Numbers near branches show the posterior probabilities from BI and bootstrap values from maximum likelihood (ML) analyses. The newly sequenced species in this study are shown in bold. The scale bar corresponds to 5 substitutions per 100 nucleotide positions. **(B)** Nucleotide differences between *Paradileptus elephantinus* and *P. conicus* based on 18S rDNA sequences. The numbers in the header indicate the unmatched site positions.

**FIGURE 7:**
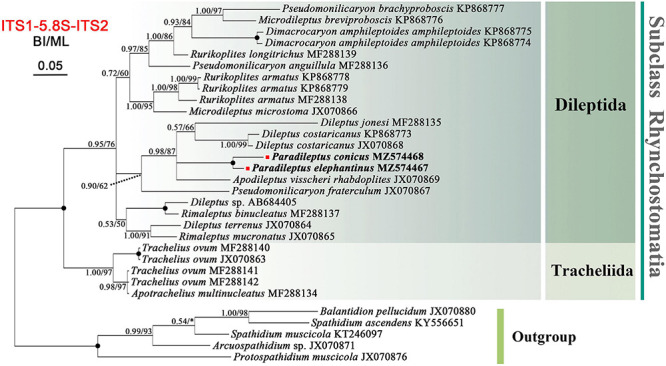
Bayesian inference (BI) tree inferred from ITS-5.8S rDNA sequences focusing on the subclass Rhynchostomatia (26 rhynchostomatian and five spathiid taxa). Numbers near branches show the posterior probabilities from BI and bootstrap values from maximum likelihood (ML) analyses. Asterisks indicate a mismatch in branching pattern between the BI and ML trees. Fully supported (1.00/100) branches are marked with solid circles. The newly sequenced species in this study are shown in bold. The scale bar corresponds to 5 substitutions per 100 nucleotide positions.

**FIGURE 8:**
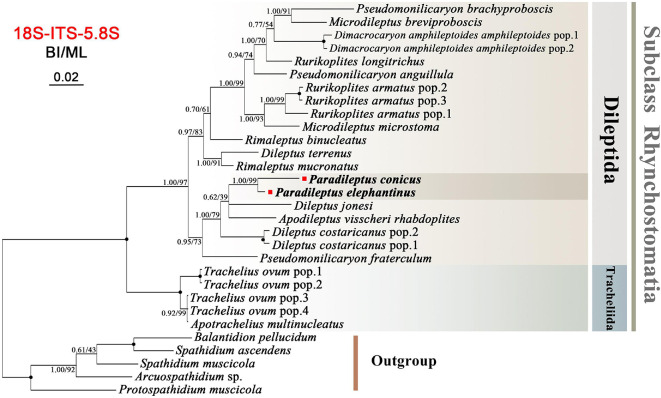
Bayesian inference (BI) tree inferred from concatenated rDNA (concatenated with 18S rDNA and ITS1-5.8S-ITS2) sequences focusing on the subclass Rhynchostomatia (25 rhynchostomatian and five spathiid taxa). Numbers near branches show the posterior probabilities from BI and bootstrap values from maximum likelihood (ML) analyses. Fully supported (1.00/100) branches are marked with solid circles. The scale bar corresponds to 5 substitutions per 100 nucleotide positions.

**FIGURE 9:**
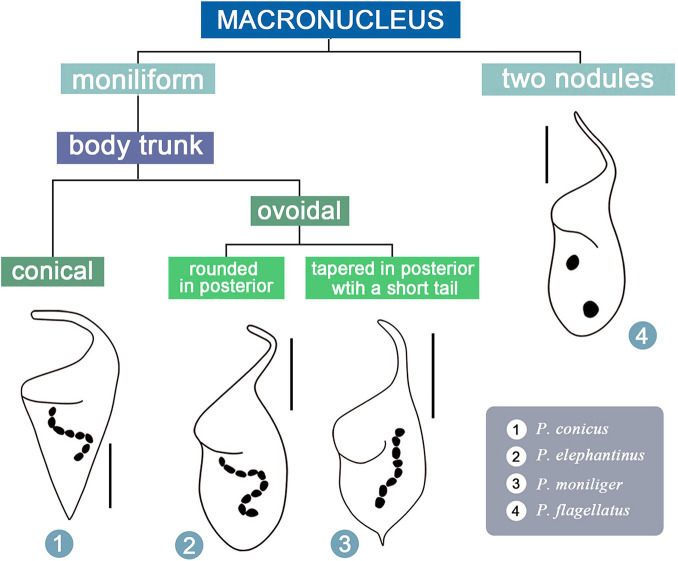
Illustrated key to valid *Paradileptus* species.

1929 *Paradileptus conicus* n. sp.–Wenrich, Transactions of the American Microscopical Society 48: 353–357 [Figures 1–4, 6–9] (original description based on living cells).
1931 *Tentaculifera mexicana* n. sp.–Sokoloff, Anales del Instituto de Biologia, Universidad Nacional Autonoma de Mexico 2: 165–166 [Figures 1, 2] (synonymy proposed by Kahl, 1935).
1935 *Paradileptus conicus* Wenrich 1929–Kahl, Die Tierwelt Deutschlands 30: 823 [Figure 20 on page 808] (brief review and description).
1943 *Paradileptus conicus* Wenrich–Kahl, Infusorien: 32 [Figure 4 on page 30] (short review).
1945 *Paradileptus conicus*–Huber-Pestalozzi, Vierteljahrsschrift der Naturforschenden Gesellschaft in Zürich 90: 120–123 [Figures 1–8 on page 125] (morphological redescription based on living cells).
1951 *Paradileptus conicus* Wenrich–Canella, Annali dell’Universita Di Ferrara (Nuova Serie) Sezione III: Biologia Animale 1: 142–148 [Figures X, 45–49 on TAV. IX] (detailed morphological redescription based on living cells).
1975 *Paradileptus conicus* Wenrich, 1929–Fryd-Versavel et al., Protistologica 11: 520–521 [Figures 16, 18C,D] (morphological redescription, including infraciliature information).
1995 *Paradileptus elephantinus* (Šveç, 1897) Kahl, 1931–Foissner et al., Informationsberichte des Bayerischen Landesamtes für Wasserwirtschaft 1/95: 203–207 [Figures 1–21] (detailed redescription based on living cells and scanning electron micrographs).
1999 *Paradileptus elephantinus* (Šveç, 1897) Kahl, 1931–Foissner et al., Informationsberichte des Bayerischen Landesamtes für Wasserwirtschaft 3/99: 221–231 [Figures 1–52] (detailed review based on living morphological characters, protargol staining, and scanning electron micrographs of a Salzburg population).
2001 *Paradileptus elephantinus* (Šveç)–Modenutti and Pérez, Brazilian Journal of Biology 61: 391 [Figure 7 on page 393] (simple redescription).
2012 *Paradileptus elephantinus* (Šveç, 1897) Kahl, 1931–Vd’ačný and Foissner, Denisia 31: 437–451 [Figures 135a–w, 136a–z, 137a–z] (valuable summary based on previous reports).

*Paradileptus conicus* has been reported many times, but some important morphological characters were still unknown prior to this study. We here present an improved diagnosis based on previous and present descriptions.

#### Improved Diagnosis

Cell size *in vivo* about 100–230 × 65–115 μm; body trunk inverted-conical; oral area with a spiral proboscis; macronucleus moniliform, micronuclei closely associated with macronuclear nodules; numerous contractile vacuoles distributed throughout body; two types of extrusomes attached to proboscis oral bulge; numerous oblong, colorless cortical granules, irregularly scattered throughout cortex; dorsal brush diffuse and staggered; about 60–85 preoral kineties; freshwater habitat.

#### Voucher Slides

Three voucher slides with protargol-stained specimens are deposited in the Laboratory of Protozoology, Ocean University of China (OUC) with registration numbers: CY2020050401-01, 02, 03.

#### Morphological Description of Chinese Population

Body size about 165–230 × 65–100 μm *in vivo*, on average about 195 × 80 μm, with length to width ratio about 2.1–2.8:1; 118–273 × 45–116 μm in protargol-stained specimens; body trunk usually inverted-conical, that is, gradually tapering from anterior end to posterior end, with large oral area at anterior trunk that extends into a helical proboscis (Figures 4A,B, 5A–E, and Table 2). Nuclear apparatus usually located in trunk; macronucleus moniliform with 6–17 nodules, about 10 nodules on average, each nodule about 17 × 11 μm in size; micronuclei not detected *in vivo* and inconspicuous in protargol-stained specimens, ovoidal or globular (about 2.6 μm in diameter, *n* = 9), closely associated with macronuclear nodules (Figures 4A,B,H, 5L,N,O). Contractile vacuoles small and numerous, about 6.9–9.5 μm in diameter, scattered beneath cell surface of trunk and proboscis (Figures 4A, 5A,D,F). Two types of extrusomes regularly arranged in proboscis oral bulge: type I rod-shaped, 4.0–5.7 μm long *in vivo*, on average about 5.0 μm; type II oblong, 1.7–2.3 μm long *in vivo*, on average about 2.0 μm; developing argentophilic extrusomes scattered throughout cytoplasm, 3.2–4.4 μm long *in vivo*, on average about 3.7 μm (Figures 4D,F, 5J,M,O). Cortex flexible with numerous oblong, colorless cortical granules (about 1.5 × 0.7 μm in size), scattered throughout cortex and thus not forming oblique rows, as usual of other dileptids (Figures 4C,E, 5G,H,K). Cytoplasm brownish at low magnifications due to cytoplasmic inclusions and dense granulation, usually with several food vacuoles containing ingested algae, without symbiotic green algae (Figures 4A, 5A–E,L). Swims moderately fast while rotating about main body axis; when disturbed, swims rapidly backward.

Somatic cilia 6–8 μm long *in vivo* and widely spaced. Somatic kineties difficult to recognize due to somatic kinetosomes (monokinetids) loosely arranged in trunk; only 1–2 recognizable somatic kineties on right side of perioral kinety, commencing at anterior end of proboscis, extending posteriad parallel to perioral kinety and terminating at trunk; somatic kinetosomes progressively loosely arranged from anterior to posterior, becoming unrecognizable near oral opening (Figures 4G–I, 5Q,S,U,W). Circumoral kinety with narrowly spaced kinetosomes, distributed along contour of oral bulge in a basically U-shaped pattern, composed of dikinetids in proboscis and monokinetids around oral opening (Figures 4G–I, 5S–Z). Perioral kinety on right of circumoral kinety with closely spaced monokinetids, commencing at anterior end of proboscis and terminating near proximal part of oral opening; space between perioral kinety and circumoral kinety narrower than that between perioral kinety and first right somatic kinety (Figures 4G–I, 5S–Z). Sixty to 85 oblique preoral kineties on left of circumoral kinety, middle kineties composed of about 10–15 narrowly spaced monokinetids, other kineties progressively shortened from middle to both ends of proboscis (Figures 4H,I, 5T,V,X,Z). Kinetosomes of dorsal brush diffuse and scattered throughout proboscis (Figures 4G–I, 5P,R). Dorsal brush also containing difficult-to-recognize monokinetidal tails with bristles about 1.9–3.4 μm long and extending to base of proboscis (Figure 5R). Type of dorsal brush bristles not discernable in protargol-stained specimens. Glabrous area on left of preoral kineties extending to proximal part of oral opening (Figures 4H,I, 5P).

Oral apparatus conspicuous, composed of a helical proboscis and a dish-like field at anterior end of trunk; oral region occupies about 42–63% of body length, distance from anterior of proboscis to oral opening about 70–125 μm (Figures 4A,B, 5A–D). Oral opening elliptical to circular, located laterally and inverted, about 20–29 × 12–23 μm in protargol-stained specimens; oral region surrounded by circumoral kinety; right side of the circumoral kinety accompanied by perioral kinety, left side associated with numerous obliquely oriented preoral kineties (Figures 4G–I, 5S–Z). Pharyngeal basket obconical, composed of numerous fibers, about 7.4–13.6 μm long *in vivo* (Figures 4F,H,I, 5H).

### Phylogenetic Analyses

The 18S rDNA sequences of the two Chinese populations were deposited in GenBank with lengths, G + C contents, and accession numbers as follows: *Paradileptus elephantinus* 1518 bp, 42.23%, MZ147012; *P. conicus* 1518 bp, 41.83%, MZ147013. The topologies of the BI and ML trees based on 18S rDNA data were highly concordant, therefore only the BI tree is presented (Figure 6A). All dileptids grouped together in the subclass Rhynchostomatia with strong support (BI 1.00, ML 99%) and were divided into two well-supported monophyletic orders, namely, Dileptida (BI 1.00, ML 97%) and Tracheliida (BI 1.00, ML 99%). The two sequences of *Paradileptus* clustered together with low support (BI 0.77, ML 23%) as a sub-clade that was sister group to *Dileptus margaritifer* (BI 0.96, ML 28%) within Dileptida. The other 18S rDNA tree focusing on the subclass Rhynchostomatia had a very similar topology (Supplementary Figure 1).

The ITS-5.8S rDNA region sequences of the two Chinese populations were deposited in GenBank with lengths, G + C contents, and accession numbers as follows: *Paradileptus elephantinus* 392 bp, 33.42%, MZ574467; *P. conicus* 391 bp, 31.71%, MZ574468. The topologies of the BI and ML trees based on ITS-5.8S rDNA data were generally concordant, therefore only the BI tree is presented (Figure 7). The phylogenetic tree inferred from ITS-5.8S rDNA data had a similar overall topology to that inferred from the 18S rDNA data, i.e., all rhynchostomatians were divided into two orders. The two *Paradileptus* species grouped together with maximal support (BI 1.00, ML 100%).

Phylogenetic reconstructions based on concatenated sequences of 18S, 5.8S, and ITS region by ML and BI methods had similar topologies; therefore, only the BI tree is presented (Figure 8). Relationships within the subclass Rhynchostomatia were generally consistent with those inferred from the single-gene analyses, the main difference being that the order Dileptida was divided into two clades instead of three, and the clustering of *Paradileptus elephantinus* and *P. conicus* was strongly supported (BI 1.00, ML 99%).

## Discussion

The genus *Paradileptus* is easily recognizable by the shape of the body, which is obliquely truncated in the oral region, and the presence of a spiral proboscis that serves to capture and manipulate prey (Wenrich, 1929; Vd’ačný and Foissner, 2012). The type species was originally reported by Rousselet (1890) as *Amphileptus flagellatus*, but Wenrich (1929) established a new genus (*Paradileptus*) for *A. flagellatus* based on the broad peristomial field with its raised rim and the spiral arrangement of the proboscis. It is noteworthy that *A. moniliger*, reported by Ehrenberg (1835), was the first species discovered in this genus, but was overlooked in subsequent revisions (Wenrich, 1929; Kahl, 1931, 1935) until it was transferred to *Paradileptus* by Vd’ačný and Foissner (2012). To date, ten nominal species of *Paradileptus* have been reported. *Paradileptus caducus* Kahl, 1935 and *P. canellai* Dragesco, 1966 are considered to be synonymous with *Pelagodileptus trachelioides* (Zacharias, 1894) Foissner et al., 1999 (Kahl, 1935; Dragesco, 1966; Foissner et al., 1999). Vd’ačný and Foissner (2012) reviewed the descriptions of the remaining eight species and concluded that only one, *P. elephantinus*, is valid. However, based on our present findings and following a reassessment of the historical studies, we consider four species to be valid, namely, *P. flagellatus* (Rousselet, 1890) Wenrich, 1929 [basionym: *Amphileptus flagellatus* Rousselet, 1890]; *P. elephantinus* (Šveç, 1897) Kahl, 1931 [see section “Results” for synonyms]; *P. conicus* Wenrich, 1929 [see section “Results” for synonyms]; and *P. moniliger* (Ehrenberg, 1835) Vd’ačný & Foissner, 2012 [synonyms: *P. ovalis* Huber-Pestalozzi, 1945; *P. estensis* Canella, 1951; *P. minutus* Dragesco, 1972; *P. elephantinus* sensu Dragesco, 1972; *P. elephantinus* sensu Dragesco and Dragesco-Kernéis, 1986; *P. elephantinus* sensu Krainer, 1988].

The type species *Paradileptus flagellatus* has only appeared in a few reports, each with inadequate illustrations (Rousselet, 1890; Wenrich, 1929; Kahl, 1931, 1943; Puytorac et al., 1972). Nevertheless, according to the original description (Rousselet, 1890), *P. flagellatus* can be distinguished from other species by having two macronuclear nodules (vs. moniliform macronucleus). The most recent report on *P. flagellatus* was that by Puytorac et al. (1972) who identified it based on observations of both living and silver-stained specimens, although no illustrations were provided. The presence of two macronuclear nodules was confirmed, thus we accept the validity of this species.

The other valid species not sampled in present work is *Paradileptus moniliger*, the body of which has a trunk that is oval in outline and tapers posteriorly to form a short tail (Ehrenberg, 1838). Populations with the same body shape have been reported several times (Huber-Pestalozzi, 1945; Canella, 1951; Dragesco, 1972a, b; Dragesco and Dragesco-Kernéis, 1986; Krainer, 1988). *Paradileptus moniliger* can be easily distinguished from its congeners by the presence (vs. absence) of a short tail (Ehrenberg, 1838; Rousselet, 1890; Šveç, 1897; Wenrich, 1929). However, according to Foissner et al. (1999), the body shape of species in this genus can quickly change in unfavorable environments with the disappearance of the tail and the body becoming more bulky. During the present study, *P. elephantinus* and *P. conicus* were starved in filtered habitat water for 3 days but there was no change in body shape. We also interchanged the living environment (filtered habitat water) of the two species, but their body shape remained unchanged after 3 days of starvation. Thus, we conclude that individuals with a short tail represent an independent species and accept the validity of *P. moniliger*.

### The Chinese Population of *Paradileptus elephantinus*

*Paradileptus elephantinus* was originally reported by Šveç (1897) under the name *Dileptus elephantinus*, but it was omitted from *Paradileptus* when Wenrich (1929) established this genus. Wenrich (1929) also described a new species, *P. robustus*, but Kahl (1935) considered this to be a junior synonym of *P. elephantinus*. The Chinese population of *P. elephantinus* closely resembles the original population with respect to its body shape, number and distribution of contractile vacuoles, moniliform macronucleus, and habitat (Šveç, 1897). The main difference is the cell size (265–420 μm in Chinese population vs. 200–250 μm). Considering the size range of the body of *P. elephantinus* (length 180–600 μm), we consider these two forms to be conspecific.

*Paradileptus flagellatus* and *P. moniliger* remain insufficiently described since there is no detailed living or infraciliature information for either. But they can still be separated from the Chinese population of *P. elephantinus* by the body shape (posterior end rounded or slightly pointed in *P. elephantinus* vs. posteriorly end sharply tapered with a short tail in *P. moniliger*) and the macronucleus (moniliform in *P. elephantinus* vs. two macronuclear nodules in *P. flagellatus*) (Ehrenberg, 1838; Wenrich, 1929).

According to the present and previous studies, *Paradileptus elephantinus* and *P. conicus* differ significantly in their morphology *in vivo* and infraciliature. The trunk of the body is oval in outline in *P. elephantinus* (vs. inverted-conical in *P. conicus*) and the extrusomes differ in length (type I, 11.6–13.4 μm and type II, 3.1–3.7 μm in *P. elephantinus* vs. type I, 4.0–5.7 μm and type II, 1.7–2.3 μm in *P. conicus*). In terms of its infraciliature, *P. elephantinus* can be distinguished from *P. conicus* by the number of preoral kineties (83–112, on average about 95 in *P. elephantinus* vs. 60–85, on average about 67 in *P. conicus*).

### The Chinese Population of *Paradileptus conicus*

*Paradileptus conicus* was first described by Wenrich (1929) who characterized it as follows: “total length usually 100–200 μm, body conical in shape, tapering posteriorly to a spike-like projection; broad anterior end occupied by a cytostome and a peristomial field, surrounded by a flange or rim from which the spirally wound proboscis arises as an extension; contractile vacuoles numerous, distributed over the body and along the posterior part of the proboscis; macronucleus beaded, composed of from four to eight segments”. The Chinese population closely resembles the original population. *Paradileptus conicus* can be clearly distinguished from *P. flagellatus* and *P. moniliger* by its body shape (inverted-conical trunk in *P. conicus* vs. ovoidal trunk with rounded posterior end in *P. flagellatus* vs. ovoidal trunk with posterior sharply tapered to form a short tail in *P. moniliger*) (Ehrenberg, 1838; Rousselet, 1890). It also can be distinguished from *P. flagellatus* by its moniliform macronucleus (vs. two macronuclear nodules) (Rousselet, 1890).

### Key to the Identification of the Four Valid Morphospecies of *Paradileptus*

For illustrations of selected key characters, see Figure 9.

(1) Two macronuclear nodules………………… *P. flagellatus*
– Moniliform macronucleus…………………………… 2
(2) Body trunk inverted-conical. . . . . . . . . . . . . . . . . . . . . . *P. conicus*
– Body trunk ovoidal………………………………… . .3
(3) Posterior end of body trunk rounded or slightly pointed ………….…….……….……………… *P. elephantinus*
– Posterior end of body trunk sharply tapered and with a short tail …………………………………… . . . . *P. moniliger*

### Molecular Phylogeny of *Paradileptus*

Rhynchostomatia was established by Jankowski (1980) as one of three subclasses of the class Litostomatea. This subclass comprises two orders, Tracheliida and Dileptida (Vd’ačný et al., 2017). Phylogenetic analyses based on 18S, 5.8S, and ITS rDNA sequence data demonstrated that each of these two orders is monophyletic (Figures 6A, 7, 8), which is consistent with previous studies (Vd’ačný et al., 2011b, 2014; Jang et al., 2014; Vd’ačný and Rajter, 2015; Huang et al., 2018).

The topology of the 18S rDNA tree (Figure 6A) shows that the two *Paradileptus* species sequenced here nest within the Dileptida where they form a clade that clusters with other dileptids in the following order: *Dileptus margaritifer* (DQ487195) followed by *Pelagodileptus trachelioides* (AB558117) followed by *Pseudomonilicaryon fraterculum* (HM581677). *Paradileptus* can be clearly separated from *D. margaritifer* by morphological features such as the body shape (wide body with a spiral proboscis in *Paradileptus* vs. narrow body with a non-spiral proboscis) and the macronucleus (moniliform or as two nodules in *Paradileptus* vs. many scattered macronuclear nodules) (Vd’ačný and Foissner, 2012). In addition, it can be distinguished from *Pseudomonilicaryon fraterculum* by the mode of locomotion (free-swimming vs. gliding in *P. fraterculum*) and the body shape (wide vs. narrow to cylindrical in *P. fraterculum*) (Vd’ačný and Foissner, 2012). *Pelagodileptus trachelioides* is a planktonic dileptid with a moniliform macronucleus that superficially resembles *Paradileptus*. However, *Paradileptus* can be distinguished by the shape of its oral opening (roundish vs. narrowly elliptical in *P. trachelioides*) and the presence (vs. absence in *P. trachelioides*) of a strongly broadened proboscis base (Foissner et al., 1999; Vd’ačný and Foissner, 2012).

Compared to the 18S tree, nodal support for the ITS-5.8S and concatenated trees was generally higher, in particular for the resolution of the *Paradileptus* clade (BI 1.00, ML 100%; BI 1.00, ML 99%). The concatenated alignment might amplify the phylogenetic signal of single markers resulting in highly resolved and robust trees for rhynchostomatians (Figures 7, 8).

### Geographical Distribution of *Paradileptus*

The findings of the present study support previous reports that suggest species of *Paradileptus*, and in particular *P. elephantinus* and *P. conicus*, are cosmopolitan (Figure 1A and Table 1; Foissner et al., 1999; Vd’ačný and Foissner, 2012). The Chinese population of *P. conicus* was isolated from an aquaculture pond where food resources are rich, whereas *P. elephantinus* was isolated from Lake Weishan where food resources are poorer. Considering that both the morphological and the 18S rDNA sequence data (Figure 6B) of these two taxa provide reliable and robust resolution of their separation at species level, we hypothesize that they might have undergone a putative speciation process *via* food preference and niche differentiation. The presence of greater numbers of preoral kineties, the larger body, the longer extrusomes and the larger buccal cavity of *P. elephantinus* suggest that its prey probably differs significantly from that of *P. conicus*, which may also be an adaptation to life in resource-poor habitats.

### Features of the Proboscis in Predatory Rhynchostomatians

Rhynchostomatians are raptorial feeders whose predatory lifestyle has led to the development of special structures for prey recognition and capture. The primary feature is the apical proboscis which carries a dorsal brush that may be used for sensory feedback, extrusomes (toxicysts) to paralyze and/or kill prey organisms, and differentiated kineties such as the circumoral kinety (∼paroral membrane) and preoral kineties (∼adoral zone of membranelles) to aid feeding (Visscher, 1923; Dragesco, 1962; Vd’ačný and Foissner, 2012; Vd’ačný et al., 2017; Chi et al., 2021). Furthermore, differences in hunting strategies and preferred prey among dileptids (Vd’ačný and Foissner, 2012) seem to be related to these differentiated structures. Exploring this phenotypic divergence might improve understanding of the distribution patterns of ryhnchostomatians and their adaptations to different environments.

According to Vd’ačný et al. (2017), the most common type of proboscis bears two kinds of extrusomes and a multi-rowed dorsal brush, which will influence speciation, extinction, and net diversification of rhynchostomatians. Therefore, we speculate that: (1) there are competitive advantages in having two types of extrusomes compared to a single type of extrusome; (2) a multi-rowed dorsal brush provides distinct advantages over a two-rowed dorsal brush by increasing sensory function during locomotion; and (3) the increased number of differentiated kineties on the proboscis improves the efficiency of predation and the dietary niche differentiation of *Paradileptus elephantinus* and *P. conicus* and could contribute to their separation as different species.

In addition to the dorsal brush, extrusomes, circumoral kinety, and preoral kineties, the rhynchostomatian proboscis also bears one or two perioral kineties, i.e., the first one or two kineties on the right side of the circumoral kinety. Most rhynchostomatians possess one perioral kinety whereas the planktonic genera *Paradileptus* and *Pelagodileptus* have two perioral kineties, which is thought to increase the efficiency of food acquisition (Foissner et al., 1999; Vd’ačný and Foissner, 2012). In the present study, we revealed that there are five to eight recognizable kineties on the right side of the circumoral kinety in *P. elephantinus*, and two to three in *P. conicus*. We also found that the first kinety to the right of the circumoral kinety has densely spaced kinetosomes, whereas those of the remaining right kineties are loosely arranged, and that the first right kinety and circumoral kinety are closely adjacent, whereas there is significantly larger gap to the second right kinety. This arrangement is found in almost all other rhynchostomatians in which the first right kinety on the right side of the circumoral kinety is the perioral kinety and the remaining right kineties are somatic kineties (for details, see Vd’ačný and Foissner, 2012). Therefore, we conclude that the first kinety to the right of the circumoral kinety in *P. elephantinus* and *P. conicus* is the perioral kinety. It is noteworthy that the arrangement of kineties on the right side of the circumoral kinety in schematic drawings of *Pelagodileptus trachelioides* is also similar to almost all other rhynchostomatians (Vd’ačný and Foissner, 2012). In particular, Packroff and Wilbert (1991) labeled to the 2nd kinety to the right of the circumoral kinety as “somatic kinety 1,” whereas the first right kinety was marked the right circumoral kinety. We suggest that this latter structure is the perioral kinety and that all rhynchostomatians are characterized by the possession of a single perioral kinety.

Finally, we speculate that the densely arranged right kineties on the proboscis (vs. loosely arranged somatic kineties in the trunk) may be a taxonomically informative character for species separation and identification in *Paradileptus*. Unfortunately, the lack of ultrastructural and ontogenetic information of the proboscis in *Paradileptus* makes the origin of these densely ciliated kineties uncertain, which should be investigated in further studies.

## Data Availability Statement

The datasets presented in this study can be found in online repositories. The names of the repository/repositories and accession number(s) can be found below: GenBank, MZ147012, MZ147013, MZ574467, and MZ574468.

## Author Contributions

YZ and HM conceived and designed the manuscript. YC carried out the live observation, protargol staining, DNA extraction, and data analyses. ZW, BL, HM, and CM checked all the data and assisted in the interpretation of the data. YC, AW, and YZ contributed to the revision of the manuscript. All authors wrote the manuscript, read and approved the final manuscript.

## Conflict of Interest

The authors declare that the research was conducted in the absence of any commercial or financial relationships that could be construed as a potential conflict of interest.

## Publisher’s Note

All claims expressed in this article are solely those of the authors and do not necessarily represent those of their affiliated organizations, or those of the publisher, the editors and the reviewers. Any product that may be evaluated in this article, or claim that may be made by its manufacturer, is not guaranteed or endorsed by the publisher.

## Footnotes

1
http://guidance.tau.ac.il/ver2/
