# Supplementary figures and images for: Taxonomy and Phylogeny of the Dileptid Ciliate Genus Paradileptus (Protista: Ciliophora), With a Brief Review and Redescriptions of Two Species Isolated From a Wetland in Northern China
Source: Front Microbiol. 2021 Sep 21;12:709566. doi: 10.3389/fmicb.2021.709566 (PMC8490868; doi:10.3389/fmicb.2021.709566)

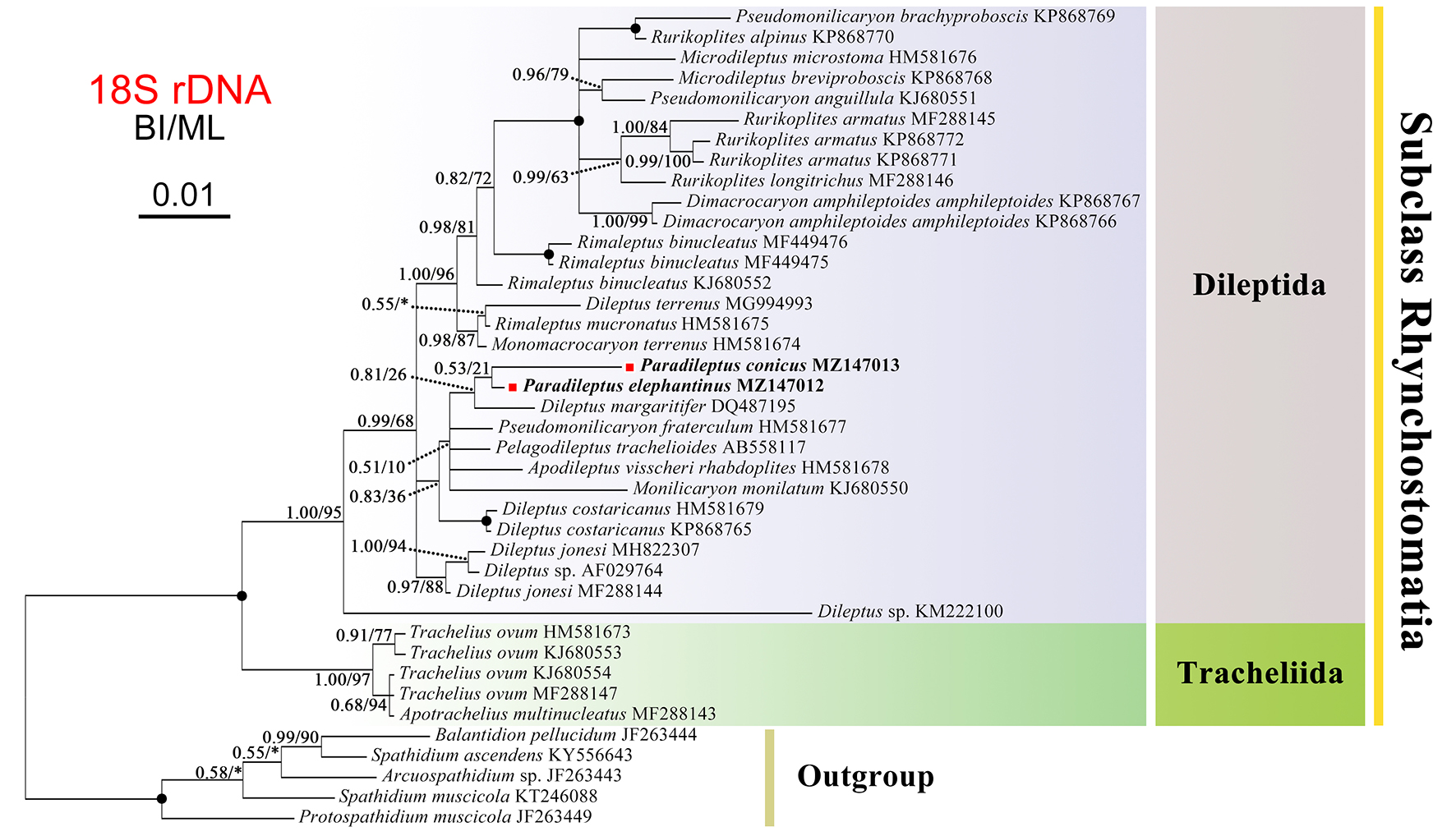

Supplement: Supplementary file 2 [file Image_1.JPEG]
